# Capgras Syndrome in Schizophrenia: A Case Report of Delusional Misidentification and Its Clinical Implications

**DOI:** 10.1192/bjo.2025.10722

**Published:** 2025-06-20

**Authors:** Duaa Fatima, Ateeq-Ur-Rehman Sheikh, Sadaf Ghulam Jelani, Palvisha Sajid, Imtiaz Ahmad Dogar

**Affiliations:** Faisalabad Medical University, Faisalabad, Pakistan

## Abstract

**Aims:** Capgras syndrome (CS) is a rare delusional misidentification syndrome characterized by the belief that a close relative has been replaced by an identical impostor. It is commonly associated with schizophrenia and other psychiatric disorders.

A 43-year-old married Asian female presented with a 5-year history of behavioural changes, including social withdrawal, suspiciousness, reduced self-care, and irrelevant speech. Her symptoms began following a familial conflict, leading to social isolation, self-neglect, and delusional misidentification, including Capgras syndrome, where she believed her mother had been replaced by an imposter. She later developed grandiose delusions, claiming to be a significant political figure. Despite initial improvement with antipsychotic treatment, she discontinued medication, resulting in symptom relapse and aggressive behaviour.

**Methods:** Case report.

**Results:** Capgras syndrome, first described in 1923, involves the delusional belief that familiar individuals have been replaced by impostors. It is often associated with psychiatric disorders, such as schizophrenia, and neurodegenerative conditions. This case aligns with the dual-route model of face recognition, suggesting impaired implicit-affective processing alongside intact conscious recognition. The patient’s aggressive behaviour underscores the potential for violence in Capgras syndrome, highlighting the need for careful risk assessment and management. Non-adherence to treatment remains a significant challenge in managing such cases.

**Conclusion:** This case highlights the importance of early recognition and sustained treatment adherence in Capgras syndrome associated with schizophrenia to prevent deterioration and improve outcomes.

